# Gallbladder cancer detected by elevated serum KL-6 levels during the follow-up of interstitial pneumonia: a case report

**DOI:** 10.1007/s13691-020-00460-0

**Published:** 2021-01-03

**Authors:** Yoshikuni Yonenaga, Manabu Kurosawa, Shunichi Higashide

**Affiliations:** 1 Department of Surgery, Nagahama City Hospital, 313 Ooinui-cho, Nagahama, Shiga 526-8580 Japan; 2 Department of Surgery, Ako City Hospital, 1090 Nakahiro, Ako, Hyogo 678-0232 Japan; 3 Department of Diagnostic Pathology, Nagahama City Hospital, Nagahama, Shiga Japan

**Keywords:** KL-6, Gallbladder cancer

## Abstract

Serum Krebs von den Lungen-6 (KL-6) is clinically used for the diagnosis of interstitial pneumonia (IP) as well as the evaluation of its disease activity. A female patient was diagnosed with idiopathic IP when she was 62 years old. Four years later, serum levels of KL-6 had been elevated gradually from 2400–3821 U/ml, and she was found to have gallbladder cancer detected by contrast-enhanced computed tomography (CT) and ^18^F-fluorodeoxyglucose (FDG)-positron emission tomography (PET)/CT. She thus underwent a radical extended cholecystectomy. After the operation, serum levels of KL-6 showed a steep decline to 590 U/ml. Immunohistological examination revealed that KL-6 presented in gallbladder cancer cells. Taken together, serum KL-6 was shown to be produced by gallbladder cancer cells. Here, we present the first patient in whom increased serum KL-6 levels led to the diagnosis of gallbladder cancer during follow-up of IP. When serum levels of KL-6 are elevated during the follow-up care of IP despite no worsening of IP, an examination of the whole body should be performed to find possibly existing malignant tumors.

## Introduction

Krebs von den Lungen-6 (KL-6) was detected by a murine monoclonal antibody which was obtained by Kohno et al. [1]. This antibody recognizes an undefined sialylated carbohydrate chain antigen on a membrane-bound glycoprotein. The molecule bearing the sialylated carbohydrate was later defined as MUC1. KL-6 was expressed by atypical and/or regenerating type II pneumocytes in tissue sections from patients with interstitial pneumonia (IP). Furthermore, Kohno et al. found that it was elevated in the serum of 70–100% of patients with IP [2]. Thus, KL-6 is useful for distinguishing most IP from other benign lung diseases, such as alveolar pneumonia, and from benign diseases of other organs. KL-6 was approved as a serum diagnostic marker for IP in Japan on Jun 1, 1999. Thus, serum KL-6 levels have been used clinically for the diagnosis of IP as well as the evaluation of its disease activity. On the other hand, Kohno et al. reported that KL-6 could not be used to distinguish a specific IP from the others or to differentiate IP from adenocarcinoma of the lung, pancreas, and breast [2]. However, adenocarcinoma of the lung, pancreas, and breast showed an almost 50% positive rate. To date, elevated serum KL-6 levels have not been suspected as an initial manifestation of gallbladder cancer. Here, we present the first patient in whom increased serum KL-6 led to the diagnosis of gallbladder cancer during the follow-up of idiopathic IP. We further investigated the expression of KL-6 antigen on gallbladder cancer cells using the immunostaining method.

## Case report

**Fig. 1:**
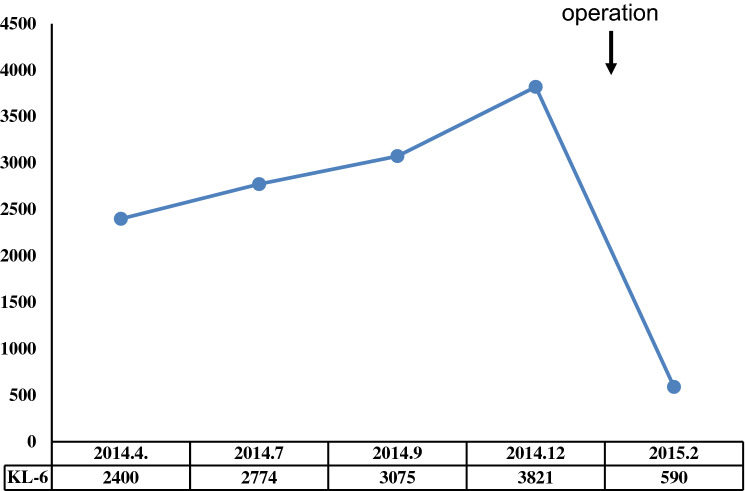
Time course of serum KL-6 levels

**Fig. 2:**
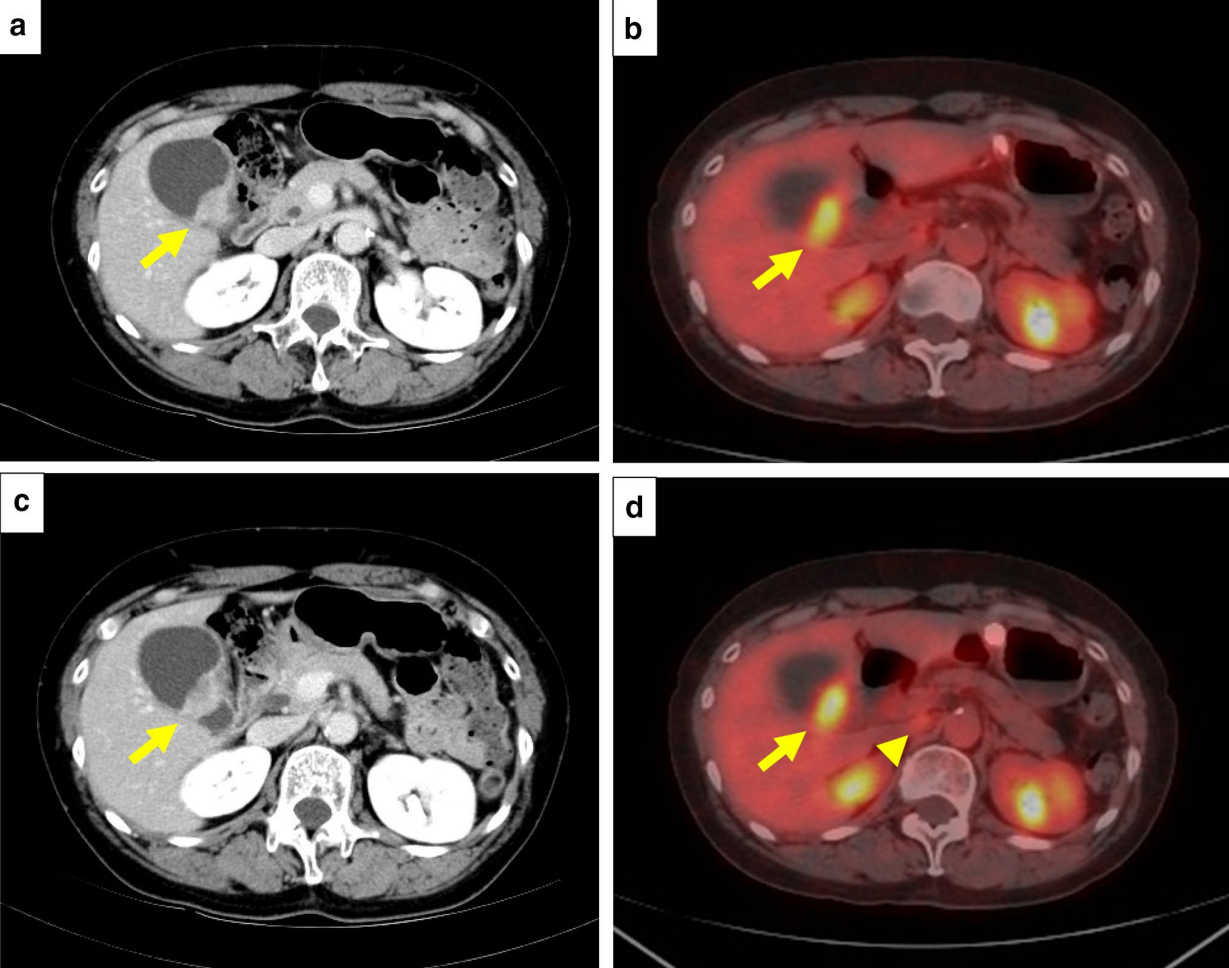
Imaging findings. **a** and **c** enhanced abdominal computed tomography (CT) showed a tumorous lesion on the body of the gallbladder (arrow). **b** ^18^F-fluorodeoxyglucose-positron emission tomography (PET)/CT showed an abnormally high uptake on the tumorous lesion (arrow). **d** The PET/CT also showed an abnormally high uptake on a regional lymph node (arrowhead), but a corresponding lymph-node swelling was not detected in the CT image (**c**)

**Fig. 3:**
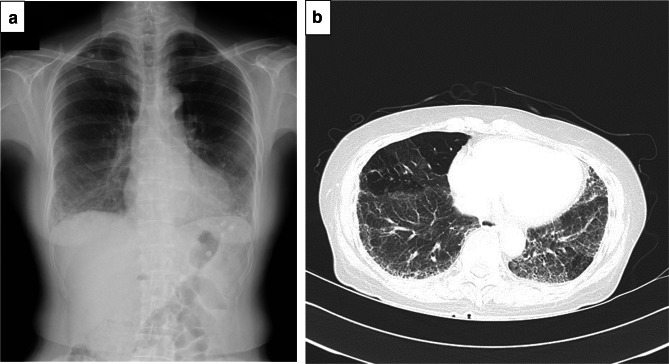
Preoperative pulmonary findings. **a** chest radiograph on admission showed ground-glass opacities in the bilateral lower lung fields. **b** chest CT showed ground-glass opacities and reticular shadows in the bilateral lung fields

**Fig. 4:**
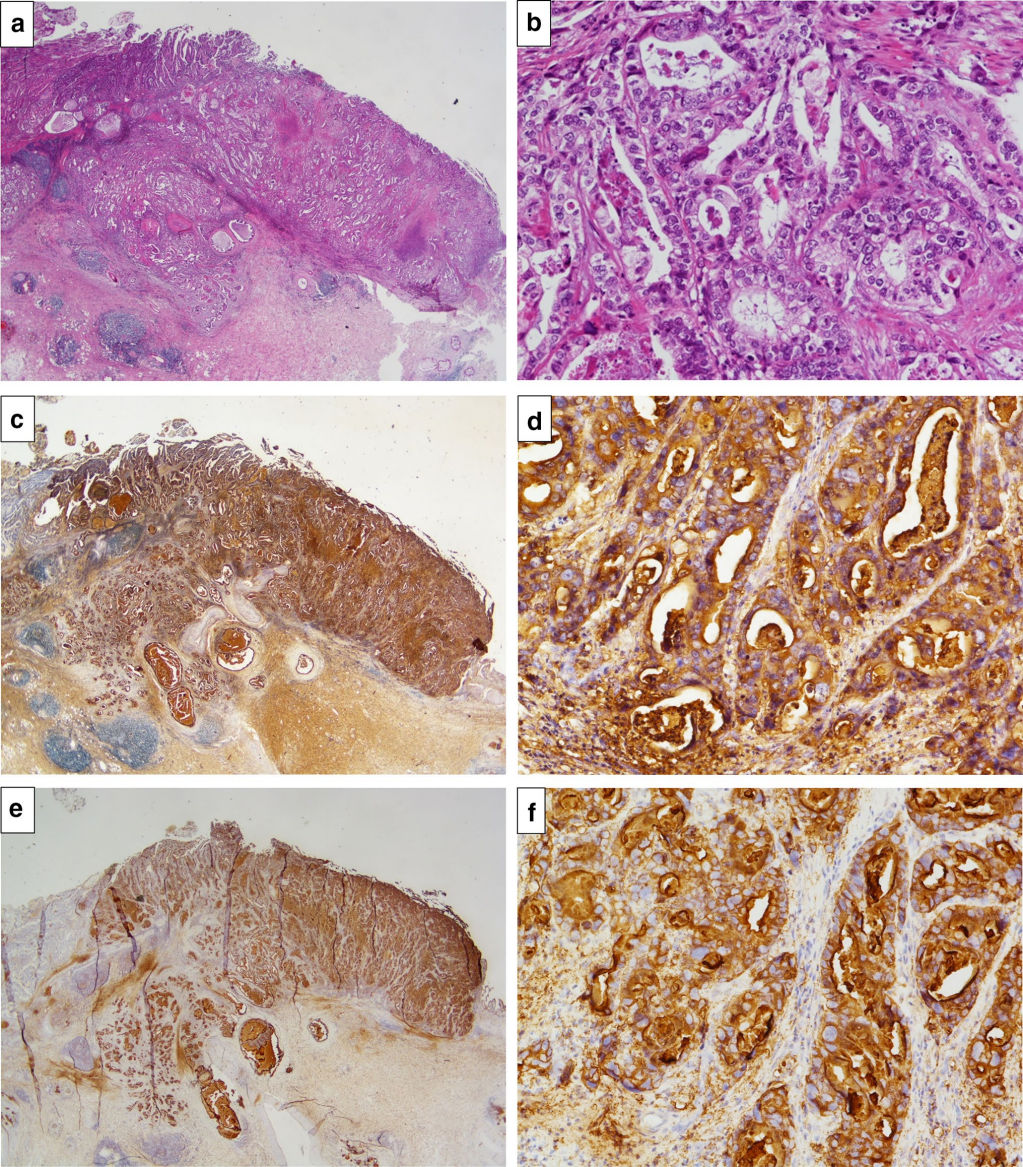
Microscopic findings. **a** and **b** the tumor represented well-to-moderately differentiated adenocarcinoma (hematoxylin and eosin staining at **a** × 10 and **b** × 200 magnification). **c** and **d** the tumor cells were ~ 80% cytoplasmic positive for KL-6 (immunostaining at **c** × 10 and **d** × 200 magnification). **e** and **f** the tumor cells were ~ 80% cytoplasmic positive for MUC1 (immunostaining at **e** × 10 and **f** × 200 magnification)

**Fig. 5:**
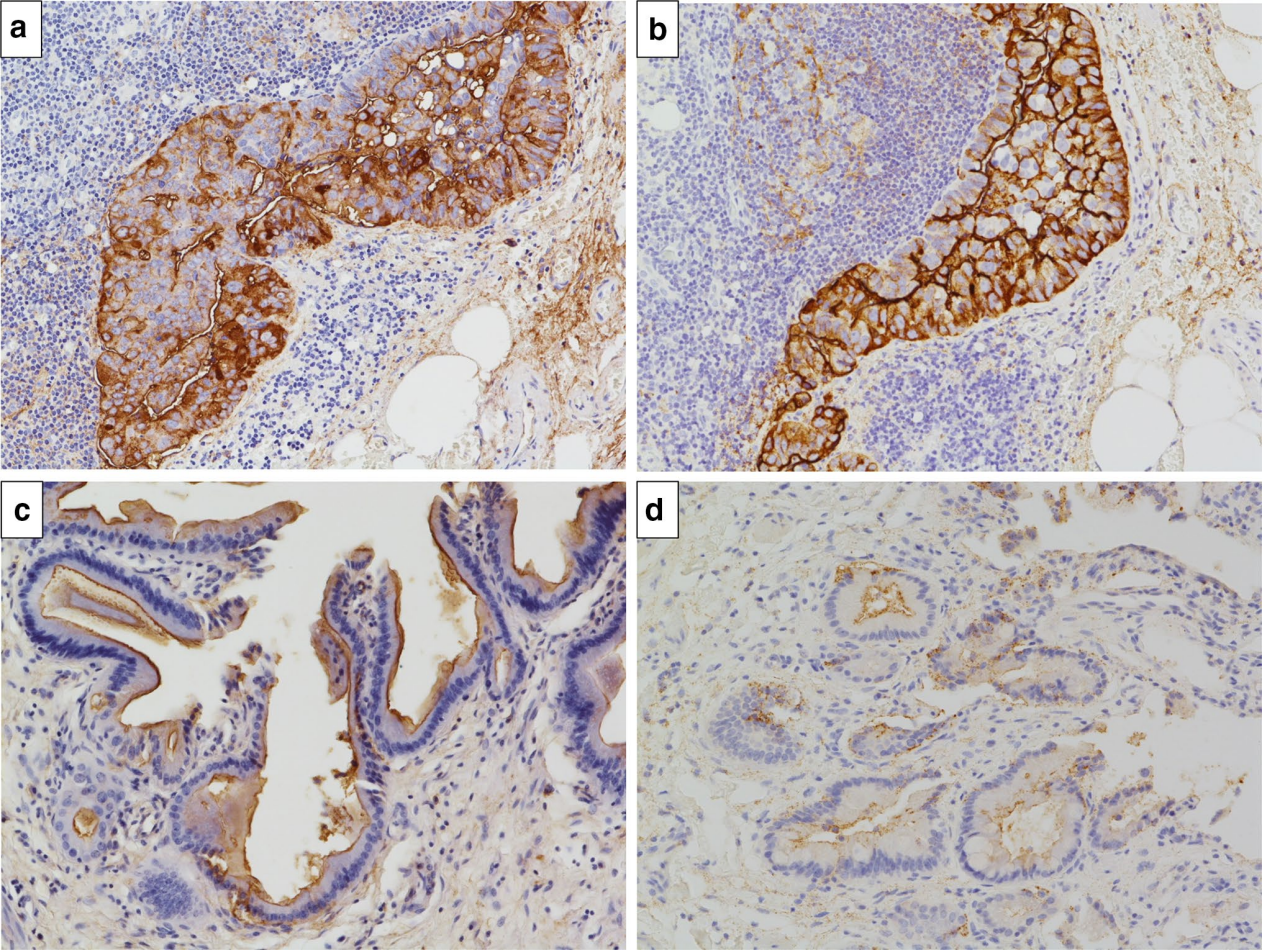
Microscopic findings. **a** The tumor cells in the metastatic lymph node were positive for KL-6 (immunostaining at × 200 magnification). **b** The tumor cells in the metastatic lymph node were positive for MUC1 (immunostaining at × 200 magnification). **c** The apical side of the noncancerous epithelia in the gallbladder was positive for KL-6 (immunostaining at × 200 magnification). **d** The apical side of the noncancerous epithelia in the gallbladder was positive for MUC1 (immunostaining at × 200 magnification)

A female patient was diagnosed with idiopathic IP in 2010 when she was 62 years old and had been followed up without therapy at our hospital. The serum KL-6 levels had been increasing gradually after Apr 2014. Specifically, values of 2400, 2774, 3075 and 3821 U/ml (normal range, < 500 U/ml) were recorded in Apr, Jul, Sep and Dec 2014, respectively (Fig. 1). However, there were no findings to indicate worsening of IP. In Sep 2014, a screening for breast cancer was performed, but nothing particular was detected. In Dec 2014, a contrast-enhanced computed tomography (CT) showed a tumorous lesion on the body of the gallbladder (Fig. 2a, c). In addition, ^18^F-fluorodeoxyglucose (FDG)-positron emission tomography (PET)/CT showed an abnormally high uptake on the tumorous lesion, with a maximum standardized uptake value (SUV_max_) of 8.1 (Fig. 2b). The PET/CT also showed an abnormally high uptake on a regional lymph node with an SUV_max_ of 2.9 (Fig. 2d), while the corresponding lymph-node swelling was not detected in the CT image (Fig. 2c). Thus, the patient was diagnosed with gallbladder cancer with a suspected lymph-node metastasis. Chest X-rays on admission showed ground-glass opacities in the bilateral lower lung fields and a chest CT taken preoperatively showed ground-glass opacities and reticular shadows in the bilateral lung fields (Fig. 3). These findings did not change during the follow-up period of IP after Apr 2014. Routine blood analysis on admission showed that complete blood counts, liver function, renal function, and electrolytes were within normal limits except for slightly elevated levels of c-reactive protein (CRP) (0.55 mg/dl; normal range, ≤ 0.5 mg/dl), alkaline phosphatase (ALP) (325 IU/L; normal range, 100–320 IU/L), and amylase (182 IU/L; normal range, 40–130 IU/L) (Table 1). The serum levels of carcinoembryonic antigen (CEA) and cancer antigen (CA)19-9 were elevated in Jan 2015 (5.0 ng/dl; normal range, < 5.0 ng/dl, 86.6 U/ml; normal range, < 37.0 U/ml, respectively), although their serum levels had been within normal limits in Sep 2014 (Table 1). Arterial blood gas analysis on admission showed slight hypoxia under ambient air (67.6 Torr; normal range, 74–108 Torr) and a slightly elevated level of bicarbonate ions (29.7 mmol/l; normal range, 21–29 mmol/l), and a pulmonary function test revealed normal vital capacity (Table 1). In Jan 2015, we performed a radical extended cholecystectomy, which consists of a cholecystectomy, wedge resection of the gallbladder bed, bile duct resection, en bloc regional lymph-node dissection, and choledochojejunostomy. She did well in the postoperative period. Formalin‑fixed paraffin‑embedded 4 μm sections of the resected specimen were used for hematoxylin and eosin staining and immunostaining. The resected specimen showed the size of the tumor was 34 × 27 mm. Pathological examination revealed that the tumor was a well-to-moderately differentiated adenocarcinoma with components of papillary adenocarcinoma and poorly differentiated adenocarcinoma (Fig. 4a, b). Two metastatic lymph nodes were identified in 12p and 13a stations according to numbers of lymph-node stations described in the Japanese classification of biliary tract cancers third English edition [3]. One of them corresponded to the abnormally high uptake shown by PET/CT preoperatively. According to the staging system described in the UICC eigth edition [4], the pathological stage of the tumor was pT2N1M1 (posterior superior pancreaticoduodenal lymph node) stage IV. Twenty-eight days after the operation, serum KL-6 levels showed a steep decline to 590 U/ml (Fig. 1). As gallbladder cancer was suspected to have caused the elevation of serum KL-6 levels, we performed further investigation by immunostaining for KL-6 (mouse anti-human KL-6 monoclonal antibody was kindly provided by Sekisui Medical, Tokyo, Japan) and MUC1 (mouse anti-human DF3 monoclonal antibody, Fujirebio Diagnostics, Tokyo, Japan). Immunohistochemical study showed that ~ 80% of the cancer cells of the primary lesion were positive for KL-6 and MUC1, with both antigens showing the same distribution pattern (Fig. 4c–f). In addition, it showed that the cancer cells of the lymph-node metastases were also positive for KL-6 and MUC1 (Fig. 5a, b). On the other hand, it showed that only the apical side of the noncancerous epithelia in the gallbladder was positive for KL-6 and MUC1 (Fig. 5c, d). Although the patient was given adjuvant chemotherapy, the levels of serum KL-6 gradually increased. A lymph-node metastasis was found in Sep 2015. At that time, the serum KL-6 level was 3511 U/ml. Liver metastasis was found in Nov 2015, lung metastasis in Aug 2016, and the patient subsequently died in Oct 2016. Because there had been no reports about KL-6 expression on the cancerous and noncancerous epithelia of gallbladders, we examined KL-6 and MUC1 expression in these areas using a further six resected specimens of gallbladder cancers from 2016 through 2019 in our hospital. The immunohistochemical localization of KL-6 and MUC1 was classified into apical and cytoplasmic types: apical type (A), KL-6, and MUC1 being restricted to the apical borders of the epithelia; cytoplasmic type (C), they being observed not only on the apical surfaces but also on the basolateral surfaces and in the cytoplasm of the epithelia [5]. KL-6 was expressed as the cytoplasmic type on more than 80% of the cancerous epithelia in all seven cases, and MUC1 was expressed as the cytoplasmic type on more than 80% of the cancerous epithelia in six out of the seven cases (Table 2). On the other hand, KL-6 was expressed as the apical type on the noncancerous epithelia in five out of six cases and MUC1 was expressed as the apical type on the noncancerous epithelia in three out of six cases (Table 2).

**Table 1:** Laboratory findings

WBC	5000 /μL
RBC	420 × 10^4^ /μl
Hb	13.0 g/dl
Ht	39.1%
PLT	27.4 × 10^4^/μl
CRP	0.55 mg/dl
AST	20 IU/L
ALT	9 IU/L
ALP	325 IU/L
γ GTP	13 IU/L
TP	7.4 g/dl
ALB	3.8 g/dl
T Bil	0.31 mg/dl
D Bil	0.08 mg/dl
CRE	0.50 mg/dl
BUN	12.9 mg/dl
AMY	182 IU/L
Na	142 mEq/L
K	4.3 mEq/L
Cl	104 mEq/L
CEA	5.0 ng/ml
CA19-9	86.6 U/ml
KL-6	3821 U/ml
Blood gas analysis (supine, room air)	
pH	7.428
PaCO_2_	45.4 Torr
PaO_2_	67.6 Torr
HCO_3_	29.4 mmol/l
SaO_2_	95.1%
Pulmonary function test	
VC	2.09 L
%VC	92.9%
FEV_1_	1.75 L
FEV_1%_	83.7%

**Table 2:** KL-6 and MUC1 expression

Case	pT	KL-6 in the cancerous epithelia	KL-6 in the noncancerous epithelia	MUC1 in the cancerous epithelia	MUC1 in the noncancerous epithelia
1	2	Positive (C)	Positive (A)	Positive (C)	Positive (A)
2	2	Positive (C)	Positive (A)	Positive (C)	Negative^b^
3	2	Positive (C)	NA	Positive (C)	NA
4	3	Positive (C)	Positive (A)	Positive (C)	Negative
5	2	Positive (C)	Positive (A), focally Positive^a^ (C)	Positive (C)	Positive (A)
6	2	Positive (C)	Positive (A)	Negative	Negative^b^
7(the current case)	2	Positive (C)	Positive (A)	Positive (C)	Positive (A)

*NA* not available. The normal epithelia scarcely remained, *C* cytoplasmic pattern, *A* apical pattern, *Negative* less than 10% positive of staining, *a* around 10% positive of staining, *b* a few positive cells identified but less than 10% positive of staining

## Discussion

We identified two important clinical issues. Elevated levels of serum KL-6 can be indicative of asymptomatic gallbladder cancer. When serum levels of KL-6 are elevated during follow-up care of IP despite no worsening of IP, an examination of the whole body should be performed to find possibly existing malignant tumors, including gallbladder cancer.

First, elevated levels of serum KL-6 can be indicative of asymptomatic gallbladder cancer. In fact, the patient had no symptoms or signs at the time of diagnosis. In this case, serum KL-6 levels showed a steep decline after the resection of the gallbladder cancer. In the study of immunostaining for KL-6, the presence of KL-6 in more than 80% of both gallbladder cancer cells and metastatic cancer cells was confirmed in the resected specimens. On the other hand, the presence of KL-6 on the noncancerous epithelia in the gallbladder was confirmed only on the apical surfaces. Kohno et al. reported that MUC1 is found at the apical surface of normal glandular epithelial cells; however, it often covers the entire cell surface in carcinomas and its level of expression is more than 10 times higher in carcinoma cells than in normal cells [2]. Taken together, serum KL-6 was shown to be produced by gallbladder cancer cells. Besides IP, Kohno et al. reported that increased KL-6 antigen levels (cut-off value 520 U/ml) were observed in the sera of patients with lung adenocarcinoma [52% (17/33)], pancreatic cancer [44% (4/9)], and breast cancer [40% (8/20)] [1]. Ogawa et al. reported that serum KL-6 was elevated (cut-off value 467 U/ml) in 31% of primary breast cancer patients [6]. Therefore, we first investigated breast cancer in this case, but moved on to further investigation when this was shown to be absent. Xu et al. reported that serum KL-6 was elevated (cut-off value 248 U/ml) in eight intrahepatic cholangiocarcinoma patients [7]. However, the cut-off value of KL-6 serum levels was lower than the usually used cut-off value of 500 U/ml. As for extrahepatic bile duct cancers, there have been no reports about their relationship with KL-6 serum levels. Although serum KL-6 is reported to be elevated in patients with several malignant tumors, there have been few case reports about cancers initially presenting with elevated serum KL-6 levels [8–10]. A case of gallbladder cancer with elevated KL-6 levels has not been reported in the English literature. This is also the first case report in the English literature comparing serum KL-6 levels in an IP patient before and after the resection of gallbladder cancer. KL-6 mucin is one kind of MUC1 mucin. Kashiwagi et al. reported on MUC1 expression in human gallbladder adenocarcinoma [11]. We also examined MUC1 expression on the gallbladder carcinoma cells, using a widely used monoclonal antibody, DF3. In this case, KL-6 and MUC1 showed the same distribution and cytoplasmic pattern. It is assumed that MUC1 may function as an anti-adhesion molecule that inhibits cell-to-cell adhesion, inducing tumor metastasis [11]. KL-6 also might function in a similar fashion.

As there have been no reports about KL-6 expression on the cancerous and noncancerous epithelia of gallbladders in the English literature, we retrospectively examined a further six cases of gallbladder cancers. The expression of KL-6 was confirmed in all cases and the expression of MUC1 was confirmed in six out of the seven. Kashiwagi et al. reported that 65% (26/40) of T2-4 gallbladder cancers showed a positive and cytoplasmic MUC1 (DF3) expression, while 7.1% (1/14) of the T1 cancers were positive [12]. In our study, the extent of six cases were pT2 and that of one case was pT3. These findings suggested that the proportion of KL-6 positivity in advanced stages would be higher than that in the early stages. Further thorough investigation is needed. In our study, KL-6 was expressed as the apical type on the noncancerous epithelia in five out of six cases and MUC1 was expressed as the apical type on the noncancerous epithelia in three out of six cases. These findings with respect to KL-6 and MUC1 localization were consistent with the previous reports about KL-6 on the renal epithelia of distal tubules and collecting ducts [13] and MUC1 on the gallbladder epithelia [5].

Second, when serum levels of KL-6 are elevated during follow-up care of IP despite no worsening of IP, an examination of the whole body should be performed to find possibly existing malignant tumors, including gallbladder cancer. In the present case, serum levels of KL-6 had risen gradually from 2400–3821 U/ml over a period of 8 months. However, the patient had shown no acute exacerbation of clinical symptoms, nor was any exacerbation evident in the findings of either chest radiograph or CT. Some cases have been reported in the English literature regarding the detection of malignancy during follow-up care of IP with no deterioration. Fukuhara et al. reported that increased serum levels of KL-6 led to the early detection of colon cancer during follow-up of dermatomyositis-associated IP [8]. Kawata et al. reported that serum levels of KL-6 increased without exacerbation of IP before liver metastasis of invasive thymoma was identified, and that the serum KL-6 level decreased following resection [9]. Kida et al. reported that an abnormally high level of KL-6 led to the diagnosis of pancreatic cancer and multiple liver metastases without progression to IP in a polymyositis patient [10]. These reports together with our case endorse our hypothesis that malignancy screening of the whole body should be performed to find possibly existing malignant tumors when serum levels of KL-6 are elevated during follow-up care of IP despite no worsening of IP.

KL-6 has been reported to be a sensitive biomarker for interstitial lung diseases in the Japanese population and has been used as a serum diagnostic marker for IP in Japan since 1999. Recently, Horimasu et al. reported that serum KL-6 levels were significantly higher in patients with interstitial lung diseases than in healthy subjects in a German cohort, although the cut-off value of KL-6 that discriminated patients with interstitial lung diseases from healthy subjects was significantly higher in the German cohort than in the Japanese cohort [14]. These findings indicate that KL-6 can be used as a diagnostic biomarker in ethnic groups other than the Japanese.

CEA and CA19-9 have been commonly used tumor markers in gallbladder carcinoma. In this case, the serum levels of CEA and CA19-9 were elevated in Jan 2015, but had been within normal limits in Sep 2014. In contrast, the levels of KL-6 had been elevated since Jul 2014. The elevation of the serum levels of KL-6 occurred much earlier than that of the serum levels of CEA or CA19-9. Thus, serum KL-6 was more sensitive than CEA or CA19-9. We retrospectively examined KL-6 expression on the cancer cells in another six cases, and the KL-6 expression was confirmed. However, the serum levels of KL-6 in these cases were not monitored in the perioperative period. Thus, much further investigation is needed. KL-6 might nonetheless prove to be a new tumor marker for gallbladder cancer in the future.

We reported a case of gallbladder cancer initially manifesting as elevated serum KL-6 levels. In conclusion, when serum levels of KL-6 are elevated during the follow-up care of IP despite no worsening of IP, screening of the whole body should be performed to find possibly existing malignant tumors, including gallbladder cancer.

## References

[1] Kohno N, Akiyama M, Kyoizumi S (1988). Detection of soluble tumor-associated antigens in sera and effusions using novel monoclonal antibodies, KL-3 and KL-6, against lung adenocarcinoma. Jpn J Clin Oncol.

[2] Kohno N (1999). Serum marker KL-6/MUC1 for the diagnosis and management of interstitial pneumonitis. J Med Invest.

[3] Miyazaki M, Ohtsuka M, Miyakawa S (2015). Classification of biliary tract cancers established by the Japanese Society of Hepato-Biliary-Pancreatic Surgery: 3(rd) English edition. J Hepatobiliary Pancreat Sci.

[4] International Union Against Cancer (UICC): TNM classification of malignant tumours, 8th ed. Brierley JD, Gopodarowicz MK, and Wittekind C, editors. Wiley-Blackwell, Chichester; 2017

[5] Kawamoto T, Shoda J, Irimura N (2001). Expression of MUC1 mucins in the subserosal layer correlates with postsurgical prognosis of pathological tumor stage 2 carcinoma of the gallbladder. Clin Cancer Res.

[6] Ogawa Y, Ishikawa T, Ikeda K (2000). Evaluation of serum KL-6, a mucin-like glycoprotein, as a tumor marker for breast cancer. Clin Cancer Res.

[7] Xu H, Inagaki Y, Tang W (2008). Elevation of serum KL-6 mucin levels in patients with cholangiocarcinoma. Hepatogastroenterology.

[8] Fukuhara N, Tanino Y, Sato S (2015). Early detection of colon cancer by increased serum level of Krebs von der Lungen-6 in a patient with dermatomyositis-associated interstitial pneumonia. Sarcoidosis Vasc Diffuse Lung Dis.

[9] Kawata T, Tsukagoshi H, Mashimo T (2002). KL-6 producing invasive thymoma. Intern Med.

[10] Kida Y, Maeshima E, Furukawa E (2007). A case of polymyositis with a significantly high level of KL-6 associated with pancreatic cancer. Mod Rheumatol.

[11] Kashiwagi H, Kijima H, Dowaki S (2001). MUC1 and MUC2 expression in human gallbladder carcinoma: a clinicopathological study and relationship with prognosis. Oncol Rep.

[12] Kashiwagi H, Kijima H, Dowaki S (2000). DF3 expression in human gallbladder carcinoma: significance for lymphatic invasion. Int J Oncol.

[13] Fukushima M, Higuchi K, Shimojo H (2012). Distinct cytoplasmic expression of KL-6 mucin in chromophobe renal cell carcinoma: a comparative immunohistochemical study with other renal epithelial cell tumors. Acta Histochem Cytochem.

[14] Horimasu Y, Hattori N, Ishikawa N (2012). Different MUC1 gene polymorphisms in German and Japanese ethnicities affect serum KL-6 levels. Respir Med.

